# The Relationship Between Preoperative Systemic Immune Inflammation Index and Prognostic Nutritional Index and the Prognosis of Patients With Alveolar Hydatid Disease

**DOI:** 10.3389/fimmu.2021.691364

**Published:** 2021-06-24

**Authors:** Bin Ren, Xiaobin Chen, Pan Lei, Lizhao Hou, Haijiu Wang, Yin Zhou, Li Ren, Haining Fan, Zhixin Wang, Jiaqi Yuan

**Affiliations:** ^1^ Department of Hepatopancreatobiliary Surgery, Affiliated Hospital of Qinghai University, Xining, China; ^2^ Key Laboratory of Hydatid Disease Research in Qinghai Province, Xining, China; ^3^ General Practice Department, Affiliated Hospital of Qinghai University, Xining, China

**Keywords:** hepatic hydatid, system immune inflammation index, prognostic factors, prognostic nutritional index, overall survival

## Abstract

**Background:**

To explore the relationship between the preoperative immune inflammation index (SII) and the prognostic nutritional index (PNI) and the overall survival rate (OS) of patients with alveolar hydatid disease.

**Methods:**

The clinical data of patients with hepatic alveolar echinococcosis treated by surgery in the Department of Hepatobiliary and Pancreatic Surgery, Affiliated Hospital of Qinghai University from January 2015 to January 2019 were analyzed retrospectively, and the SII, PNI, PLR and NLR were calculated. Spearman correlation analysis was utilized to analyze the correlation among SII, PNI, PLR and NLR. Receiver operating characteristic curve (ROC) was utilized to determine the best intercept values of SII, PNI, PLR and NLR, and Chi-square test was used to evaluate the relationship between SII, PNI and various clinicopathological features in patients with hepatic alveolar echinococcosis. The kaplan-Meier method was used to draw survival curves and analyze the relationship between them and the total survival time of patients. A cox regression model was used to analyze the relationship between SII, PNI and the prognosis of patients with hepatic alveolar echinococcosis. Finally, ROC curve was used to estimate the predictive efficacy of SII, PNI and COSII-PNI for the prognosis of patients with hepatic alveolar echinococcosis.

**Results:**

A total of 242 patients were included, including 96 males and 146 females, aged 11.0-67.0 (36.6 ± 11.7) years. The values of SII, PNI, PLR and NLR are calculated, and the best truncation values of SII, PNI, PLR and NLR are given in ROC curve. The kaplan-Meier survival curve was used to analyze the relationship between SII, PNI, PLR, NLR and the overall survival time of patients with hepatic alveolar echinococcosis. The results showed that the median follow-up time was 45 months (95%CI: 39.484-50.516), and the average survival time was 49 months (95%CI: 47.300-51.931), which was low p<0.001); The 5-year OS rate of low PNI was significantly lower than that of high PNI group (37.7% *vs* 71.6%; p<0.001); The 5-year OS rate in low PLR group was significantly higher than that in high PLR group (70.4% *vs* 24.3%; p<0.001); The 5-year OS rate in low NLR group was significantly higher than that in high NLR group (67.2% *vs* 28.8%; p<0.001). Cox unifoliate analysis showed that SII, PNI, PLR and NLR were important prognostic factors related to OS. Cox multivariate analysis showed that SII(HR=4.678, 95% CI: 2.581-8.480, P<0.001) and PNI(HR=0.530, 95%CI: 0.305-0.920, P<0.05) were identified as independent risk indicators of OS, while NL was identified as independent risk indicators of OS ROC curve analysis showed that AUC of SII, PNI, PLR, NLR and COSII-PNI were 0.670(95%CI: 0.601-0.738), 0.638(95%CI: 0.561-0.716) and 0.618(95% CI: 0.541-0.694), respectively COSII-PNI is superior to SII and PNI in evaluating prognosis (P < 0.05).

**Conclusions:**

SII and PNI can be regarded as independent risk factors reflecting the prognosis of patients with hepatic alveolar echinococcosis. The lower SII and the higher PNI before operation, the better the prognosis of patients, and the combined application of SII and PNI before operation can improve the accuracy of prediction.

## Introduction

Echinococcosis is a kind of zoonotic parasitic disease caused by Echinococcus larvae, which is mainly divided into two types: one is echinococcosis caused by Echinococcus granulose larvae infection, also known as cystic echinococcosis; The other is echinococcosis caused by Echinococcus multiculturalism larvae infection, also known as alveolar echinococcosis (1, 2). Echinococcus can spread to the liver through portal circulation, and a few can cause echinococcosis in other organs such as heart, lung, kidney and brain through the lungs, about 70% of which occur in the liver and 20% in the lungs (3). The incidence of hepatic alveolar echinococcosis is slightly lower than that of cystic echinococcosis. However, due to its growth characteristics similar to malignant tumor (easy to invade adjacent organs and distant metastasis through blood vessels, lymphatic vessels and biliary tract), it has potent pathogenicity and high fatality rate. At present, the treatment of hepatic hydatid disease is mainly surgical treatment, supplemented by chemotherapy and other comprehensive treatments. However, because hydatid disease grows slowly and its early symptoms are not evident, most patients miss the best treatment stage, resulting in poor surgical results and poor prognosis. Therefore, it is extremely important to explore the effective prediction of the prognosis of patients with hepatic alveolar echinococcosis and make individualized treatment plan.

In recent years, related studies have confirmed that the inflammatory reaction plays an important role in all stages of the occurrence and development of hepatic hydatid disease, which involves the changes of Th1 and Th2, Th17 and Treg cell-related pro-inflammatory and anti-inflammatory factors. Considering the imbalance of cell function (4–6). It is useful to noting that more and more scholars pay more attention to the inflammatory indicators that can reflect the whole body, such as platelet/lymphocyte ratio (PLR), lymphocyte/neutrophil ratio (NLR) and lymphocyte/monocyte ratio (LMR) (7, 8). However, this inflammation-based biomarker integrates only two kinds of immune cells. Recent studies have shown that systemic immune inflammatory, SII Index (SII), a comprehensive index based on platelets, lymphocytes and neutrophils in peripheral blood, can more comprehensively reflect the balance between immune status and host inflammation, and has obvious advantages in predicting prognosis. It has been shown to be pancreatic cancer, breast cancer, gastric cancer, gallbladder cancer, lung cancer and liver cells (9–11).

In recent years, related studies have shown that nutritional status and immune function play an important part in the occurrence and development of tumors. Patients’ nutritional status and immune function are linked to the choice of treatment and the quality of life. Therefore, monitoring the nutritional status and immune status of the body has certain guiding significance for the curative effect and prognosis (12). Prognostic nutritional index (PNI), which was first proposed by Buzby et al. (13) in 1980, is mainly calculated by counting serum albumin and peripheral blood lymphocytes, and can comprehensively reflect the nutritional status and immune status of patients (14–16). Flavill et al. (17) first proposed its role in preoperative nutrition, immune function and surgical risk assessment of gastrointestinal cancer patients. Contemporary studies have shown that PNI is closely related to the prognosis of colorectal cancer, hepatocellular carcinoma and esophageal cancer (18–20).

## Methods

This study was agreed by the Institutional Research Ethics Board of Qinghai University Affiliated Hospital and successfully registered in Chinese Clinical Trial Registry (No.PSL2018006). All methods were performed in accordance with the Declaration of Helsinki, and this study did not involve human or animal tissue or blood samples, and all patients signed the written informed consent before surgery.

### Patients

The clinical data of 242 patients with hepatic alveolar echinococcosis treated by hepatomegaly and pancreatic surgery in Affiliated Hospital of Qinghai University from January 2015 to January 2019 were analyzed retrospectively. Surgical methods include radical liver hydatid surgery and palliative treatment. Inclusion criteria: (1) Diagnosis of hepatic alveolar echinococcosis by abdominal B ultrasound and abdominal CT; (2) Before operation, there was not any targeted application of albendazole anti-insect drugs. (3) Those who did not have acute and chronic inflammation before operation and had normal blood routine examination results; (4) Child-Pugh grade of preoperative liver function is A or B; Exclusion criteria: (1) The postoperative pathological diagnosis is not hepatic alveolar echinococcosis; (2) Patients with missing or missing medical records.

### Assessment of SII and Other Inflammation-Based Prognostic Scores

Collect the first blood collection results of patients in hospital, and calculate SII, PNI, PLR, NLR, SII =PxN/L; PNI=ALB(g/L)+5xL(10^9^/L); PLR=P/L; NLR=N/L; In which P, N, L and M are peripheral platelets, neutrophils, lymphocyte counts and monocytes respectively. They were divided into groups according to the optimal critical value, and the relationship between them and clinical pathological factors was analyzed.

### Follow Up

All postoperative patients were monitored regularly. The follow-up period was once every 3-6 months, and the follow-up was done by outpatient service, SMS, telephone and WeChat. Follow-up included the current general situation of patients, whether or not hepatic hydatid recurred and the time of recurrence, whether or not there were adjuvant treatment and treatment plan after operation, and the time and cause of death of dead patients. The total survival time was identified as the time from the first day after operation to death or follow-up deadline, and the follow-up deadline was May 2020 or the patient died.

### Statistical Analysis

SPSS22.0 and Graph Pad Prism8.0 were used to analyze the data statistically. The counting data were expressed by n(%), and χ^2^ tests were used for comparison between groups. Spearman correlation analysis was used to analyze the correlation among SII, PNI, PLR and NLR. Receiver operating characteristic curve (ROC) was utilized to determine the best cut-off values of SII, PNI, PLR and NLR. According to the cut-off values, patients were divided into 2 groups: low and high. A chi-square test was used to evaluate the relationship between SII, PNI, PLR and NLR and various clinicopathological features in patients with hepatic alveolar echinococcosis. The kaplan-Meier method was used to draw the survival curve, and log-rank test was used to compare the differences between the two groups. A cox regression model was used for university and multivariate analysis, and the risk ratio and corresponding 95% confidence interval (CI) were calculated. The difference was statistically significant at P<0.05.

## Results

### Patients’ Characteristics

After screening, a total of 242 patients met the criteria, including 146 women (60.3%) and 96 men (39.7%), median ages 38 (28-44) years. All 242 patients were diagnosed as hepatic alveolar echinococcosis, and were discharged smoothly after operation, with no death. Among all patients, 187 cases (77.3%) received radical treatment of hepatic hydatid disease and 55 cases (22.7%) received palliative treatment. As of May 31, 2020, all 242 patients were followed up with a median follow-up time of 45 months, of which 70 cases (28.9%) died and 172 cases (71.1%) were still alive.

### Determination of Optimal Critical Values of SII, PNI, PLR, and NLR Before Operation

Spearmen correlation analysis showed that PNI was negatively correlated with NLR (r=0.497, P<0.001). PNI was negatively correlated with PLR (r=0.472, p< 0.001). There is a negative correlation between PNI and SII (r=0.348, p<0.001). Therefore, our findings confirmed that PNI is associated with the systemic inflammatory response markers in HAE. We determined the best cut-off values of these biomarkers by ROC analysis to predict OS 5 years after surgery. The area under OS curve (AUC) is 0.670(95%CI: 0.601-0.738), 0.638(95%CI: 0.561-0.716), 0.642(95%CI: 0.568-0.715),and 0.618(95%CI: 0.541-0.684) of SII, PNI, PLR and NLR, respectively Taking the value corresponding to the maximum Youden index as the cutoff value, the corresponding optimal cutoff values are SII 758.92, PNI 42.275, PLR 178.145 and NLR 2.695. According to the cutoff value, the patients were divided into two groups for further analysis: low SII group (SII ≤ 758.92, n=126) and high SII group (SII>758.92, n=116); Low PNI group (PNI ≤ 42.275, n=134) and high PNI group (PNI>42.275, n=108); Low PLR group (<178.145, n=114) and high PLR group (≥178.145, n=128); Low NLR group (<2.695, n=128) and high NLR group (≥2.695, n=114).

### Patient Clinicopathological Characteristics and Their Associations With SII

SII was related to operation mode, blood loss, Child-Pugh grade, complications, ALT, AST, TBil, ALB, ALP, PT, platelet and leukocyte count (P<0.05), but not to age, sex, hydatid metastasis, number of lesions, neutrophil and PMN stage (P>0.05) PNI was related to operation mode, intraoperative blood loss, PNM stage, hydatid metastasis, focus number, Child-Pugh grade, AST value, TBil value, ALB value, ALP value, PT value and lymphocyte count (P<0.05). It was not related to age, sex, complications, ALT value, neutrophil, platelet and leukocyte count (P>0.05). PLR was related to operation mode, intraoperative blood loss, lesion number, Child-Pugh grade, complications, ALT, AST, TBil, ALB, ALP, PT, platelets and lymphocytes (P<0.05), but not to age, sex, hydatid metastasis, neutrophil and leukocyte count (P> 0.05) NLR was related to gender, surgical method, intraoperative blood loss, PMN stage, Child-Pugh grade, ALT, AST, TBil, ALB, ALP, PT, neutrophil, lymphocyte and leukocyte count (P<0.05), but not to age, hydatid metastasis, number of lesions, complications and platelet count (P >0.05), as showed in **Table 1**.

**Table 1:** Correlations between the SII, NLR, PLR, PNI, and clinicopathological variables in patients with HAE.

Variables	PNI			SII			NLR			PLR		
				
	High	Low	P	High	Low	P	High	Low	P	High	Low	P
	n=108	n=134		n=116	n=126		n=114	n=128		n=128	n=114	
Age			0.157			0.130			0.372			0.824
≤30	39	37		42	34		38	38		46	35	
>30	69	97		74	92		76	90		87	79	
Sex			0.242			0.511			<0.001			0.516
Male	36	60		43	53		59	36		48	48	
Female	72	75		73	73		55	92		80	66	
Surgical approach			<0.001			<0.001			<0.001			<0.001
Radical treatment	96	91		75	112		74	113		86	101	
Palliative care	12	43		41	14		40	15		42	13	
Intraoperative blood loss			<0.001			<0.001			<0.001			<0.001
<1000ml	77	57		50	92		50	92		56	86	
≥1000ml	23	85		66	34		64	36		72	28	
PNM stages			<0.001			0.040			0.063			<0.001
I II	70	45		47	68		45	70		48	67	
≥III	38	89		69	58		69	58		80	47	
Whether to transfer			<0.001			0.207						0.028
Yes	76	115		95	96		93	98		21	10	
None	32	19		21	30		21	30		108	83	
Lesion number			<0.001			0.693			0.349			0.035
Single	79	66		68	77		63	82		69	76	
Multiple	29	68		48	49		51	46		59	37	
Child–Pugh			<0.001			<0.05			<0.001			<0.001
A	68	36		38	66		27	77		41	63	
B	40	98		78	60		87	51		87	51	
complication			0.062			0.003			0.009			<0.001
Yes	51	80		90	41		72	60		84	48	
None	57	54		26	85		42	68		44	66	
ALT(U/L)			0.369			<0.001			<0.001			0.024
≤40	53	58		43	68		37	74		50	61	
>40	55	76		73	58		77	54		78	53	
AST(U/L)			0.041			0.01			0.002			0.021
≤40	61	58		48	71		44	75		54	65	
>40	47	76		68	55		70	53		74	49	
TBil(μmol/L)			<0.001			0.021			<0.001			<0.001
≤32.4	75	58		52	81		40	93		54	79	
>32.4	33	76		64	45		74	35		74	35	
ALB(g/L)			<0.001			0.002			0.004			<0.001
≤35	32	126		84	74		85	73		98	60	
>35	76	8		32	52		29	55		30	54	
ALP(U/L)			<0.001			0.01			<0.001			<0.001
≤150	36	18		18	36		10	44		17	37	
>150)	72	116		98	90		104	84		111	77	
PT(s)			<0.001			0.02			<0.001			0.002
≤16	68	43		43	68		39	72		47	64	
>16	40	91		73	58		75	56		81	50	
NE			0.805			0.231			<0.001			0.663
≤6.3	100	123		104	119		95	128		117	105	
>6.3	8	11		12	7		19	0		11	8	
PLT			0.684			<0.001			0.843			<0.001
≤300	56	73		44	85		60	69		46	83	
>300	52	61		72	41		54	59		82	31	
LY			0.002			0.031			<0.001			<0.001
≤0.8	1	14		13	2		14	1		14	1	
>0.8	107	120		103	124		100	127		114	113	
WBC			0.854			0.06			0.007			0.385
≤10	92	113		93	112		85	116		106	99	
>10	16	21		23	14		25	12		22	15	

### Relationship Between SII, PNI, PLR, NLR, and Overall Survival Time of Patients With Hepatic Alveolar Echinococcosis

**Figure 1:**
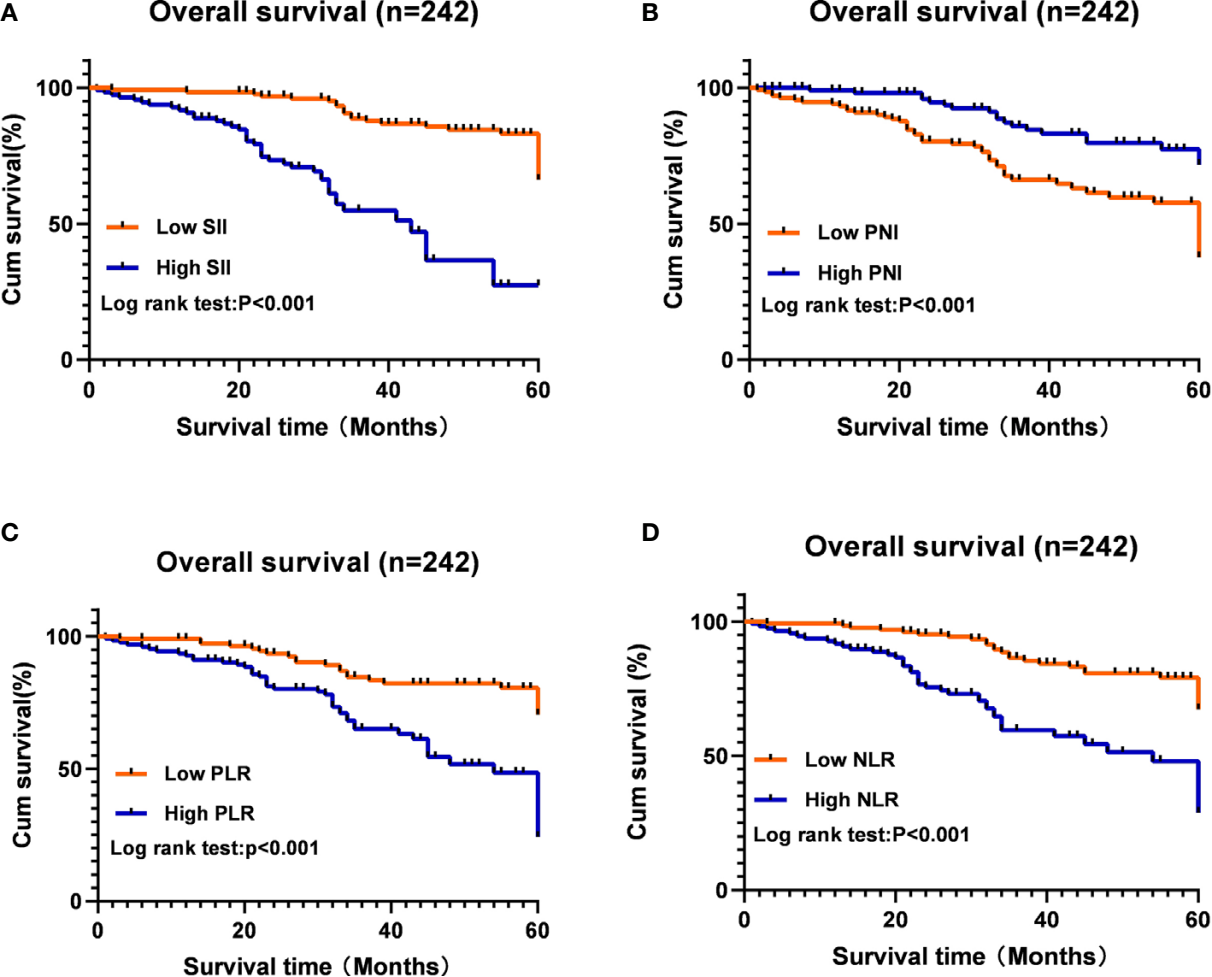
Kaplan Meier survival curves for OS according to inflammation-based scores in hepatic hydatid patients. **(A)** patients with SII > 758.92 × 109/L had worse prognosis than patients with SII ≤758.92 × 109/L (P < 0.001). **(B)** patients with PNI > 42.275 had longer OS than patients with PNI ≤ 42.275 (P < 0.001). **(C)** patients with PLR > 178.145 had shorter OS than patients with PLR ≤ 178.145 (P < 0.001). **(D)** patients with NLR > 2.695 had worse prognosis than patients with NLR ≤ 2.695 (P < 0.001).

The median follow-up time was 45 months (95%CI: 39.484-50.516), and the average survival time was 49 months (95%CI: 47.300-51.931). The correlation between inflammatory biomarkers and OS is shown in **Figure 1**. The 5-year OS rate in low SII group was significantly higher than that in high SII group (66.1% *vs* 27.4%; p<0.001; **Figure 1A**); The 5-year OS rate of low PNI was significantly lower than that of high PNI group (37.7% *vs* 71.6%; p<0.001; **Figure 1B**); The 5-year OS rate in low PLR group was significantly higher than that in high PLR group (70.4% *vs* 24.3%; p<0.001; **Figure 1C**); The 5-year OS rate in low NLR group was significantly higher than that in high NLR group (67.2% *vs* 28.8%; p<0.001; **Figure 1D**).

### Univariate and Multivariate Analysis of Influencing Survival of Patients

Univariate analysis showed that treatment mode, PNM stage, SII, PNI, PLR and NLR were important prognostic factors related to OS. The multivariate Cox regression model showed that SII(HR=4.678, 95%CI: 2.581-8.480, P<0.001), PNI(HR=0.530, 95% CI: 0.305-0.920, P<0.05), treatment mode (HR=1.910, 95%CI: 1.146-3.182, P<0.05) was determined as an independent risk index of OS, while PNM stage, NLR and PLR were not independent risk factors of OS, as shown in **Table 2**.

**Table 2:** Univariate and multivariate Cox proportional hazards regression models for overall survival in patients with HAE.

	Univariate analysis		Multivariate analysis	
	HR (95% CI)	P	HR (95% CI)	P
Gender				
Male	1			
Female	1.008 (0.988 1.028)	0.439		
Age				
≤31	1			
>31	1.272 (0.744 2.175)	0.379		
treatment				
Radical treatment	1		1	
Palliative care	3.19(1.973 5.176)	0.000	1.91(1.146 3.182)	0.013
Far metastasis				
yes	1			
no	1.43(0.731 2.797)	0.296		
PNM stages				
I,II	1		1	
III	1.96(1.184 3.257)	0.009	0.911 (0.512 1.622)	NS
SII				
Low	1		1	
High	5.907 (3.386 10.306)	0.000	4.678(2.581 8.480)	0.000
PNI				
Low	1		1	
High	0.368 (0.217 0.625)	0.000	0.530(0.305 0.920)	0.024
NLR				
Low	1		1	
High	3.328 (2.036 5.440)	0.000	1.493(0.830 2.687)	NS
PLR				
Low	1		1	
High	3.111 (1.859 5.206)	0.000	1.169 (0.610 2.242)	NS

### Prognostic Value of COSII-PNI in Patients With Hepatic Alveolar Echinococcosis After Operation

**Figure 2:**
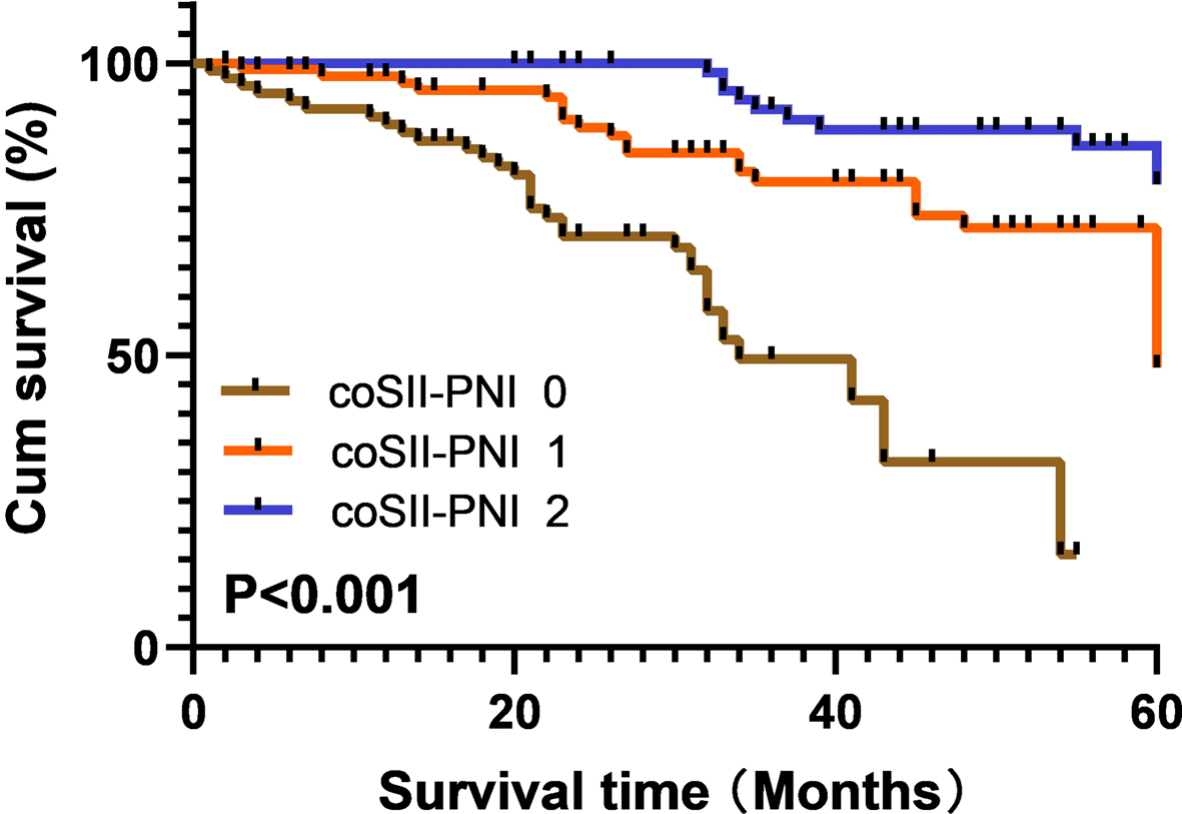
Kaplan–Meier survival curves for OS according to the combination of SII and PNI in hepatic hydatid patients. The 5-year OS rates for patients with coSII-PNI = 0, 1, and 2 were 23.5%, 47.6%, and 79.3%, respectively (p < 0.001).

**Figure 3:**
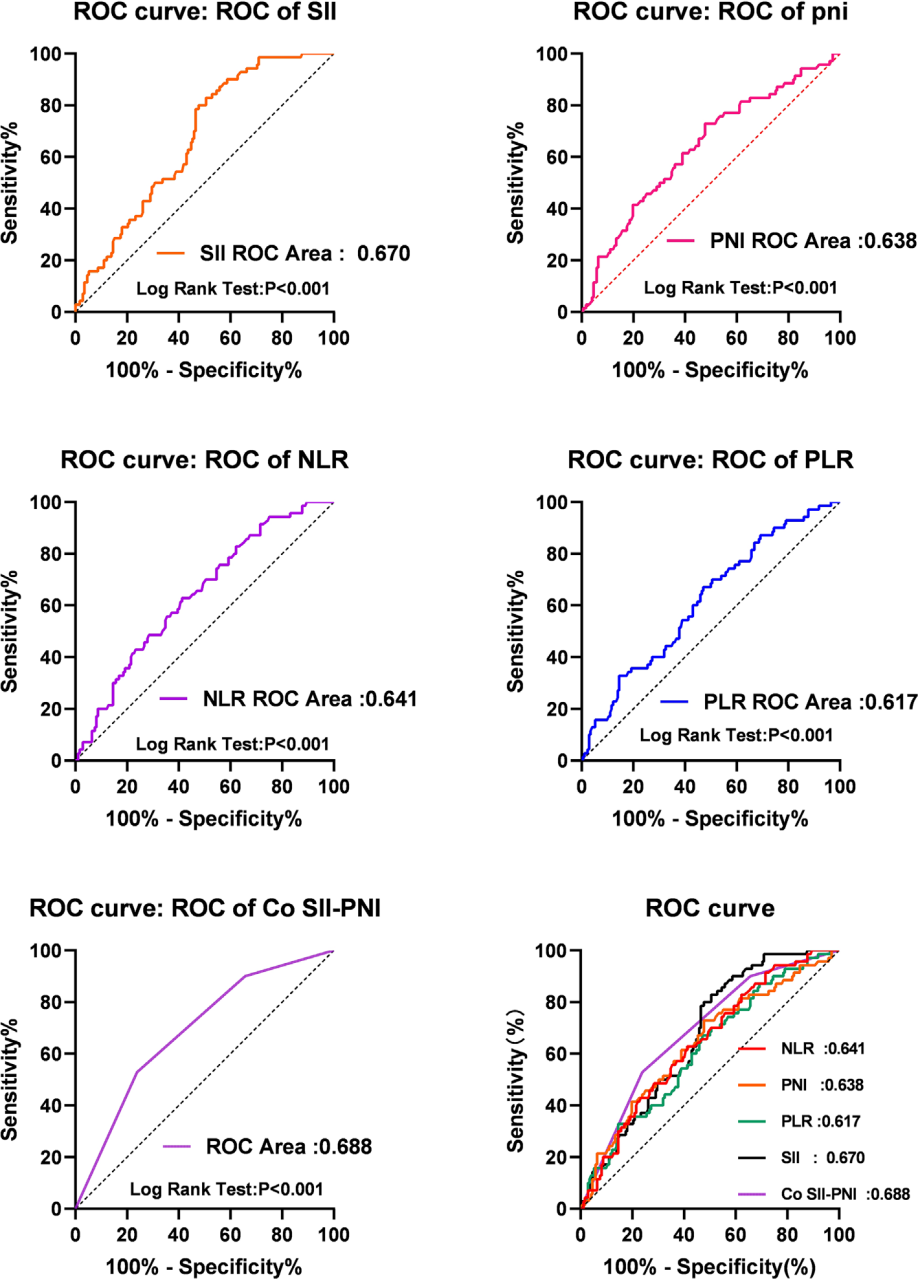
The AUC of SII, PNI, PLR, NLR and COSII-PNI were 0.670(95%CI: 0.601-0.738), 0.638(95%CI: 0.561-0.716), 0.642(95%CI: 0.568-0.715),0.618(95%CI: 0.541-0.684) and 0.688(95%CI: 0.616-0.760) and respectively Compared with SII or PNI alone, we found that COSII-PNI has the highest AUC, which indicates that COSII-PNI is the most accurate prognostic indicator for predicting survival rate among these inflammatory indicators, and can be used as a tool for evaluating the prognosis of patients with hepatic alveolar echinococcosis.

Finally, we evaluated the prognostic value of COSII-PNI in patients with hepatic alveolar echinococcosis after treatment. Patients with low SII and high PNI scored 2 points, patients with high SII and high PNI or low SII and low PNI scored 1 point, and patients with high SII and low PNI scored 0 point. Kaplan-Meier analysis and log rank test showed that the 5-year survival rates of patients with COSII-PNI=0, 1 and 2 were 23.5%, 47.6% and 79.3%, respectively, as shown in **Figure 2**. Through ROC curve analysis, we compared the prediction accuracy of SII, PNI, PLR, NLR and COSII-PNI for 5 years OS. The AUC of SII, PNI, PLR, NLR and COSII-PNI were 0.670(95%CI: 0.601-0.738), 0.638(95%CI: 0.561-0.716), 0.642(95%CI: 0.568-0.715),0.618(95%CI: 0.541-0.684) and 0.688(95%CI: 0.616-0.760) and respectively Compared with SII or PNI alone, we found that COSII-PNI has the highest AUC, which indicates that COSII-PNI is the most accurate prognostic indicator for predicting survival rate among these inflammatory indicators, and can be used as a tool for evaluating the prognosis of patients with hepatic alveolar echinococcosis. as shown in **Figure 3**.

## Discussion

In recent years, more and more studies have shown that inflammation plays a major role in the occurrence, development and metastasis of diseases, and also affects the host’s immune regulation (18, 21). In our study, we investigated the clinical and prognostic value of preoperative systemic inflammatory markers (including SII, PNI, PLR and NLR) in patients with hepatic alveolar echinococcosis, and compared their predictive accuracy. Our results demonstrate that the preoperative high-level SII and low-level PNI are independent predictors of postoperative OS in patients with hepatic alveolar echinococcosis. As far as we know, this is the first time that to compare the prognostic value of these four preoperative inflammatory indicators in patients with hepatic alveolar echinococcosis.

In this study, we first reported the clinical and prognostic value of SII and PNI in patients with hepatic alveolar echinococcosis. Firstly, we determine the best intercept values of SII, PNI, PLR and NLR by using receiver operating characteristic curve (ROC), and divide them into high and low groups. By analyzing the relationship between SII and clinicopathological features of patients, we found that SII before operation was related to operation mode, interpretative blood loss, Child-Pugh grade, complications, ALT, AST, TBil, ALB, ALP, PT, platelet and leukocyte count (P< 0.05). Preoperative PNI was related to the operation mode, interpretative blood loss, PNM stage, hydatid metastasis, focus number, Child-Pugh grade, AST value, TBil value, ALB value, ALP value, PT value and lymphocyte count (P<0.05). Preoperative PLR was related to the operation mode, interpretative blood loss, lesion number, Child-Pugh grade, complications, ALT, AST, TBil, ALB, ALP, PT, platelets and lymphocytes (P<0.05). The preoperative NLR was related to gender, operation mode, interpretative blood loss, PMN stage, Child-Pugh grade, ALT, AST, TBil, ALB, ALP, PT, neutrophil, lymphocyte and leukocyte count (P<0.05).

In our study, unifoliate analysis confirmed that except SII and PNI, treatment methods, PNM stages, PLR and NLR were significantly correlated with OS. In multivariate analysis, SII, PNI and treatment methods were independent prognostic indicators of OS in patients with hepatic alveolar echinococcosis, which had important clinical significance. As far as we know, this is the first time that SII and PNI have been combined to evaluate the prognosis of patients with hepatic alveolar echinococcosis.

By investigating the relationship between preoperative SII and the prognosis of patients with hepatic alveolar echinococcosis, we found that the survival time of patients with high SII was significantly shortened, and the 5-year survival rate was significantly lower than that of patients with low SII (27.4% *vs* 66.1%, P<0.05). Higher SII mostly represents higher levels of granulocytes and platelets and lower levels of lymphocytes, which fundamentally mean that the immune response of patients with hepatic alveolar echinococcosis is weakened and the inflammatory response is enhanced, resulting in a poor prognosis. Therefore, a better explanation of the role of neutrophils, platelets and lymphocytes in the process of parasite infection is useful to explain the relationship between SII and the prognosis of patients with hepatic alveolar echinococcosis. Neutrophils, as an important cellular component of the body’s defense against infection, also play an important protective role in parasitic infection, killing invading parasites mainly through ADCC effect and phagocytosis (22). Platelets interact with IgE through the receptor of specific IgE with low affinity on its surface membrane, and this IgE-dependent platelet activity can lead to parasite killing (23). NF-κB and TGF-β/Smad pathway are activated by platelet-derived transforming growth factor (TGF-β1) in liver tissue of patients with hepatic hydatid disease. Thus inducing mesenchyme transition and promoting metastasis sympathetically. TGF-β1 down-regulates the expression of NK cell activity receptor NKG2D through a series of ways, which eventually lead to the weakening of NK cell killing protoscolex and the formation of immune escape from host. Therefore, platelets play an important role in the metastasis and survival of worms (24). When initial patients are infected with echinococcosis, lymphocytes can induce the secretion of related cytokines and participate in the body’s immune system to kill Echinococcus, and inhibit the growth and distant metastasis of the worm. Subsequently, hepatic echinococcosis can induce the expression of PD-1 on the surface of liver-specific CD4^+^ and CD8^+^ effector T cells by secreting antigens and using surface molecules. And through PD-1/PD-L1 pathway, the number of T cells is reduced, the proliferation ability and the secretion of cytokines (such as IL-2, IFN-γ, IL-10) are inhibited, which leads to the immune escape of hepatic alveolar echinococcosis and enables it to survive in the body for a long time (25). The decrease of lymphocyte count leads to the weakening of host immunity, which reflects the inhibitory state of the host immune system. These may be the main reasons for the poor prognosis of patients with high-level SII hepatic alveolar echinococcosis.

By analyzing the correlation between preoperative PNI and prognosis, we found that the survival time of patients with hepatic alveolar echinococcosis with low PNI was significantly shortened, and the 5-year survival rate was significantly lower than that of patients with high PNI (37.7% *vs* 71.6%; p<0.001). Considering the influence of PNI on the prognosis of patients with hepatic alveolar echinococcosis may include the following aspects: on the one hand, the liver is the main place for synthesizing albumin, and albumin, as the main component of plasma protein, is an important substance in human body, can reflect the nutritional status of patients, is an independent influencing factor on the prognosis of patients, and is also an antidote and transporter, which has been widely used to evaluate the progress of diseases and predict the survival of patients (26). IL-1, IL-6 and TNF-α, which are highly expressed in the serum of patients with hepatic alveolar echinococcosis, can inhibit the synthesis of ALB, which also leads to hypoalbuminemia (4); On the other hand, lymphocytes play an important role in immune monitoring and immune mediation. The decrease of lymphocytes represents the immunosuppressive state of host, which can lead to a poor prognosis. Therefore, the combined PNI can reflect the immune and nutritional status of patients with hepatic alveolar echinococcosis at the same time, which may be the main reason for the poor prognosis of patients with hepatic alveolar echinococcosis with low level PNI.

In this study, we reviewed the clinical and prognostic value of preoperative systemic inflammatory markers (including SII, PNI, PLR and NLR) in patients with hepatic alveolar echinococcosis. The results showed that SII and PNI were independent prognostic factors, but SII or PNI alone had low accuracy in telling the prognosis of patients with hepatic alveolar echinococcosis. Therefore, we further evaluated whether the combined application of SII and PNI could improve the prognostic value of patients with hepatic alveolar echinococcosis. Our data demonstrate that patients with low SII and high PNI have the best prognosis, while patients with low PNI and SII have the worst prognosis. ROC curve analysis shows that COSII-PNI has the highest AUC, which indicates that COSII-PNI can predict the prognosis of patients with hepatic alveolar echinococcosis more accurately than SII or PNI alone. These results all support our hypothesis that the combined application of SII and PNI can improve the prognosis accuracy of patients with hepatic alveolar echinococcosis.

Our experiment has some limitations. First of all, this is a retrospective study with a small sample, and there may be selection bias in the process of data collection. Second, this study does not include the evaluation of additional inflammatory indicators, such as C- reactive protein, α1- acid glycoprotein, interleukin and so on (27). Thirdly, in some studies, the cutoff values of SII are not consistent, and most of them are considered dependable, so it is impossible to determine the ideal cutoff value, which limits the practical application of SII in clinic. Fourthly, the related mechanism of neutrophils, lymphocytes and lymphocytes affecting hepatic alveolar echinococcosis is not clear, and it is not clear whether high SII before operation means promoting or inhibiting peripheral neutrophils, lymphocytes and platelets. Therefore, in the future, we still will need to conduct a larger-scale forward-looking study to confirm our preliminary results. With the further development of advanced research, SII can help medical staff to choose the operation and treatment plan.

In a word, our experimental results show that SII and PNI, as new, accurate and objective biomarkers for biological prediction, are expected to be indicators for evaluating the prognosis of patients with hepatic alveolar echinococcosis because of their advantages of simplicity, convenience in calculation, universality, non-invasive and low price, and the combined application of SII and PNI can improve the prediction accuracy of 5-year OS of patients with hepatic alveolar echinococcosis.

## Data Availability Statement

The original contributions presented in the study are included in the article/supplementary material. Further inquiries can be directed to the corresponding authors.

## Ethics Statement

The studies involving human participants were reviewed and approved by the Ethics Committee of the Affiliated Hospital of Qinghai University, Grant No.PSL2018006. The patients/participants provided their written informed consent to participate in this study. Written informed consent was obtained from the individual(s), and minor(s)’ legal guardian/next of kin, for the publication of any potentially identifiable images or data included in this article.

## Author Contributions

BR, XC, and PL: Designed the research and wrote the paper. JY and ZW: Revised the paper. LH and HF: Participated in research work. BR: Collected samples. JY, ZW, and BR: Analyzed data and constructed figures. All authors contributed to the article and approved the submitted version.

## Funding

This work was supported by National Key R&D Program Project (2017YFC0909900), Qinghai Provincial Science and Technology Department Project (2020-ZJ-Y01).

## Conflict of Interest

The authors declare that the research was conducted in the absence of any commercial or financial relationships that could be construed as a potential conflict of interest.
